# Correction: A polypropylene mesh coated with interpenetrating double network hydrogel for local drug delivery in temporary closure of open abdomen

**DOI:** 10.1039/d5ra90092a

**Published:** 2025-08-04

**Authors:** Ze Li, Changliang Wu, Zhen Liu, Zhenlu Li, Xingang Peng, Jinjian Huang, Jianan Ren, Peige Wang

**Affiliations:** a Department of Emergency Surgery, The Affiliated Hospital of Qingdao University 16 Jiangsu Road Qingdao 266000 P. R. China wpgzyz@163.com; b Lab for Trauma and Surgical Infections, Department of Surgery, Jinling Hospital, Affiliated to Southeast University 305 East Zhongshan Road Nanjing 210002 P. R. China 220163109@seu.edu.cn Jiananr@gmail.com

## Abstract

Correction for ‘A polypropylene mesh coated with interpenetrating double network hydrogel for local drug delivery in temporary closure of open abdomen’ by Ze Li *et al.*, *RSC Adv.*, 2020, **10**, 1331–1340, https://doi.org/10.1039/C9RA10455K.

The authors regret an error in Fig. 7A of the published article. The correct Fig. 7 is as shown here. An independent expert has viewed the corrected figure and has concluded that it is consistent with the discussions and conclusions presented in the article.

**Fig. 7:**
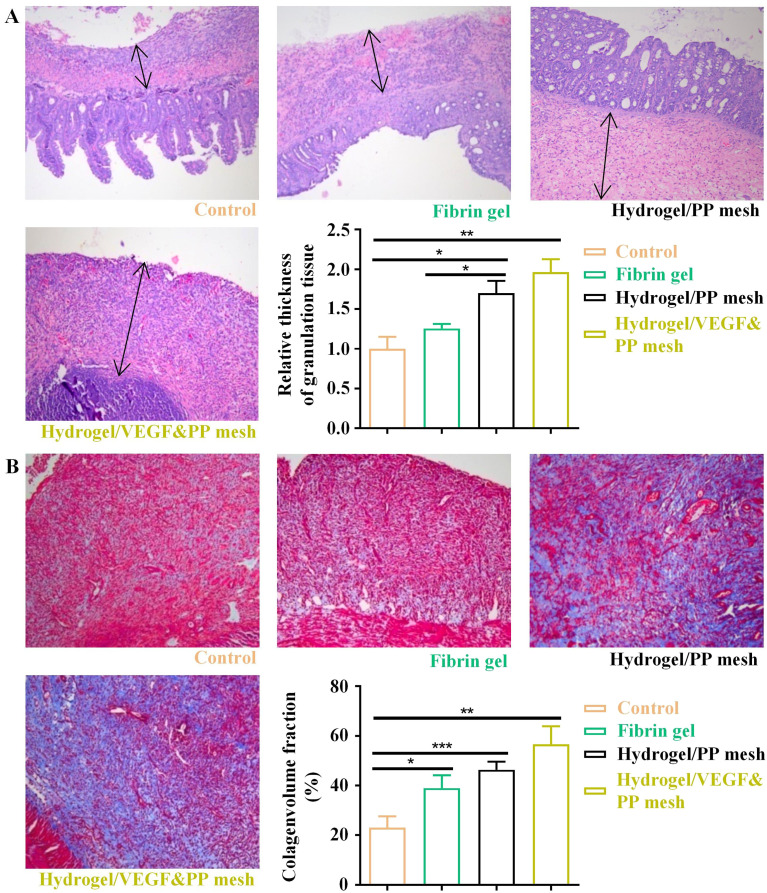
Assessment of four potential treatments on protection of abdominal wall defect of rat models. (A) HE staining of regenerative abdominal wall tissues (blank arrows: regenerative tissues), 10×. *P < 0.05, **P < 0.01. (B) Masson staining of regenerative abdominal wall tissues, 10×. *P < 0.05, **P < 0.01, ***P < 0.001.

The Royal Society of Chemistry apologises for these errors and any consequent inconvenience to authors and readers.

